# A replication study in dendrochronology—revisiting the panels of two portraits of Rembrandt

**DOI:** 10.1057/s41599-025-06066-2

**Published:** 2025-11-19

**Authors:** Marta Domínguez-Delmás

**Affiliations:** 1 Department of Archaeology, Cultural Heritage Agency of the Netherlands, Smallepad 5, 3811MG Amersfoort, The Netherlands; 2 Functional Traits Group, Naturalis Biodiversity Center, Darwinweg 2, 2333CR Leiden, The Netherlands

**Keywords:** Cultural and media studies, Philosophy

## Abstract

In the Replicating a Rembrandt Study project, which revisited the attribution of two portraits of Rembrandt while exploring the strengths and limitations of replication studies in art history, dendrochronological research was carried out to reproduce and replicate research conducted in the 1990s. One of the portraits, at the Germanisches Nationalmuseum in Nuremberg, Germany, had then been attributed to Rembrandt, whereas the other one, at the collections of the Mauritshuis in The Hague, The Netherlands, was considered a copy. In this study, the reproduction involved reassessing the results obtained for the two panels in the 1990s by comparing the tree-ring measurements produced then with reference oak chronologies from the source area (Poland and the eastern Baltic), and with a third panel from the Rijksmuseum that had matched the wood of the Nuremberg portrait, and seemingly originated from the same oak tree. The replication entailed remeasuring the tree rings in the panels through digital photography and using modern software to compare them to new reference chronologies and the Rijksmuseum panel. Both approaches confirmed the original results, including the southern Baltic provenance of the wood and a same-tree match between the Nuremberg and Rijksmuseum panels. However, the reproduction identified measurement errors in the initial study, while the replication corrected these errors. Furthermore, thanks to the improved reference datasets currently available, the replication provided more accurate interpretations about the felling dates of the trees. This research demonstrates that dendrochronology is a reliable science that should yield consistent results if applied rigorously, regardless of the software or ring-width acquisition method employed. However, for reproducibility, detailed reporting, including reference datasets used and statistical values obtained, is required. Long-term storage of dendrochronological data and digital images from the tree-ring sequences allows for verification and reanalysis without the need to re-examine the artworks. Therefore, it is advised that museums and art collectors commissioning dendrochronological research request dendrochronological reports that contain detailed graphs and information, as well as the shared stewardship of the tree-ring datasets and digital images produced by dendrochronologists.

## Introduction

**Fig. 1:**
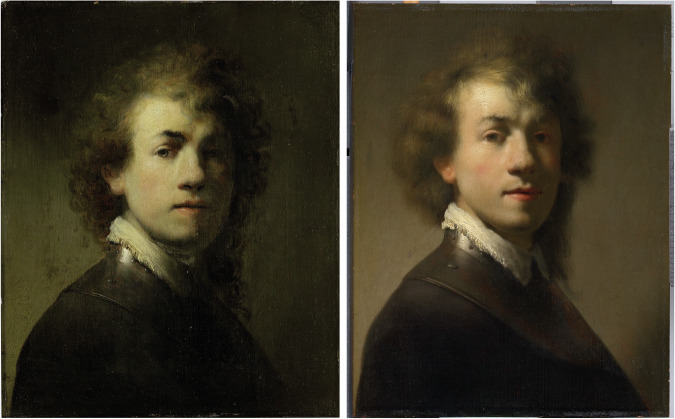
Portraits of a young Rembrandt. Left, GMN version: portrait at the Germanisches Nationalmuseum in Nuremberg, Germany (GM391, *Self-Portrait with gorget*; oil on panel, H38.2 × W31 cm, https://rkd.nl/technical/5008997), attributed to Rembrandt van Rijn; right, MH version: portrait at the Mauritshuis in The Hague, the Netherlands, currently considered a studio copy (Mh148, *Portrait of Rembrandt (1606–1669) with a Gorget*; oil on panel, H37.9 × W28.9 cm, https://rkd.nl/technical/5005287). Source: RKD technical.

The *Replicating a Rembrandt Study* project was carried out at the Vrije Universiteit of Amsterdam in close collaboration with the Mauritshuis (MH) museum in The Hague (the Netherlands) from 2022 to 2024. This project explored the strengths and limitations of replication studies in the humanities by carrying out a replication study within the field of art history (Rulkens et al., 2025). The replicated study concerned an investigation published in 1999 and 2000, about the attributions of two portraits of Rembrandt in the collections of the Mauritshuis in The Hague (MH, inventory number 148) and the Germanisches Nationalmuseum in Nuremberg (GNM, inventory number 391) (Buijsen^ 1999^; Wadum, 1998) (Fig. 1). The GNM version is signed by Rembrandt and dated art historically ca. 1629. The MH painting is accepted as a studio copy of one of Rembrandt’s pupils, and is dated based on stylistic similarity to the GNM painting.

As part of the original research in the 1990s, dendrochronology was carried out by Prof. Dr. Peter Klein from the University of Hamburg to date the panels. Therefore, the aim of the replication study presented here was to find out whether the dates reported by Klein in the 1990s (i.e., date of the outermost ring in each board, estimated felling date of the trees, and earliest possible production date of the paintings) could be replicated in two ways: first by carrying out a so called *reproduction*, i.e., using the data produced by Klein and the same study protocol to assess the results from the 1990s; and second, by a *conceptual replication* which allows for a modified study protocol. i.e., using current state-of-the-art methods to measure the tree rings and comparing them with new reference datasets.

## Brief retrospective of the 1990s dendrochronological study and results

### Klein’s description of the panels and reported dates

The reports and tree-ring series produced by Klein in the 1990s were obtained for this study from the Netherlands Institute for Art History (RKD) through RKDTechnical and the Dendro4Art database.^1^ According to the reports, both paintings are made of single boards of oak (*Quercus* sp.). The panel of the GNM version (Klein’s dendro-code 4001501A) was reported to have 210 rings and to lack sapwood, but it was found to match a panel from the Rijksmuseum collection in Amsterdam that ends in the same heartwood ring and has eight sapwood rings (https://rkd.nl/technical/5008997; Table 1). The one from the MH panel (Klein’s dendro-code 4004301A) was reported to have 101 rings and to also lack sapwood (https://rkd.nl/technical/5005287). The last (outermost) ring in the GNM panel was dated to 1606 CE, and that of the MH panel to 1602 CE. Klein reported for both panels that the wood had a Baltic origin, without specifying the reference chronologies he had used.

**Table 1:** Dendrochronological results as reported by Klein in the 1990s.

Painting	Current attribution	Klein’s dendro-code	*N*	Begin date	End date	Earliest possible felling date (more likely)^c^	Earliest possible production date (more likely)	Source area wood
Germanisches Nationalmuseum Nuremberg (GNM)^a^	Rembrandt van Rijn	4001501A	210	1397	1606	From 1615 (1619/1621/1625)	From 1617 (1623)	Baltic
Mauritshuis The Hague (MH)^b^	Rembrandt’s studio copy	4004301A	101	1502	1602	From 1611 (1615/1617/1621 + x)	From 1613 (1619)	Baltic

*N*: number of measured rings.

^a^https://rkd.nl/explore/technical/5008997.

^b^https://rkd.nl/explore/technical/5005287.

^c^Estimations based on sapwood statistics reported by Wazny (1990).

Once the exact date of the outermost ring present in the wood of the panel was determined, Klein proceeded to estimate the felling dates of the trees. While his report lacks an explanation about how he came up with the estimated felling dates, he mentions the use of “sapwood statistics for Eastern Europe”. Based on the resulting estimations, it becomes apparent that he used the sapwood statistics for Poland published by Wazny (1990), which were the only sapwood estimates available for Eastern Europe at the time. These statistics indicate that oak trees growing in Poland have between 9 and 24 sapwood rings (or 15 −6/+9) within a 90% confidence interval (Haneca et al., 2005). Considering this, Klein wrote: “[…] the earliest possible felling date of the tree used is from 1615, though a felling date between 1619…1621…1625 is more likely”. The *earliest felling date* for the tree in 1615 results from adding the lower end of the estimation interval (9 sapwood rings) to the date of the last ring present in the panel, 1606 (Table 1). The more likely felling date in 1621 results from adding the median point of the estimation interval (15 rings) to the 1606 date, but an explanation for the 1619 and 1625 estimations cannot be found in the report. Following the same rationale, he added 9 years to the end date of the MH panel (1602) to propose an *earliest possible felling date* for the tree in 1611 CE, and added “though a felling date between 1615…1617…1621 + x is more likely”. Again, the 1617 results from adding 15 to the last measured ring to reach the median of the estimation interval, and the “+ x” is used to indicate that there are rings still missing to the heartwood/sapwood boundary (indicating therefore that it is a *terminus post quem*). However, the 1615 and 1621 estimations are not explained in the report. Lastly, accounting for the seasoning time of the wood, Klein added 2 more years to both dates and presented them as the *earliest possible production date of the painting* (1613), and the *more likely production date of the painting* (1619) (Table 1).

### The match of the GNM panel with a Rijksmuseum painting

**Fig. 2:**
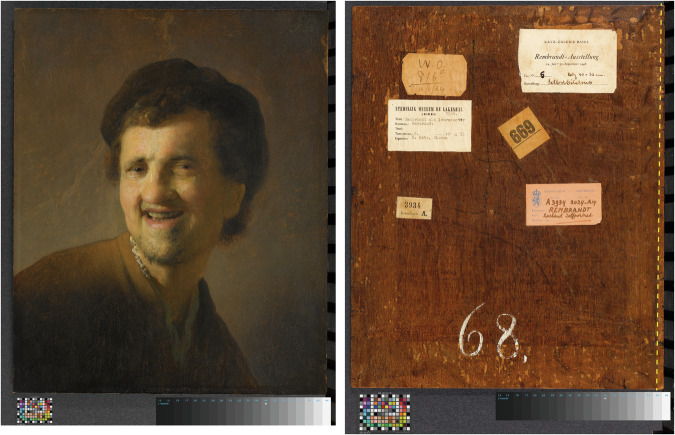
Front and back of *Laughing young man*, attributed to the circle of Rembrandt van Rijn (oil on panel, h 41.2 cm × w 33.8 cm × d 4 cm; Rijksmuseum inventory nr. SK-A-3934). The dashed line runs along the heartwood/sapwood border. Source: https://rkd.nl/technical/5009945.

In addition to the date for the GNM painting at the Germanisches Nationalmuseum, Klein also reported that the board originates from the same tree as the board of the painting *Laughing young man* at the Rijksmuseum, Amsterdam, attributed to the circle of Rembrandt (RM, inventory number SK-A-3934) (Fig. 2). In his report about the RM painting, Klein reported that the panel has 231 rings, including 8 sapwood rings, and that the outermost ring dates to 1614 CE (https://rkd.nl/technical/5002600). Based on these observations, he argued in the report of the GNM panel that the outermost ring (dating to 1606) is the ring corresponding to the heartwood/sapwood border.

## Material and methods

### Reproduction of the original dendrochronological research

The first part of the replication of the dendrochronological study carried out in the 1990s consisted of examining the tree-ring series obtained by Klein to determine whether the same dates could be obtained with chronologies from the area reported by Klein as the source area of the wood. Then, the tree-ring series of the GNM panel was compared to that of the RM painting to verify Klein’s observations about the panels deriving from the same tree.

#### Reference chronologies

Dendrochronology was established in Europe in the 1940s. In the 1970s, wood scientists of Hamburg University (where Klein would develop his career) carried out the first dendrochronological studies on Netherlandish panel paintings (Bauch et al., 1972; Eckstein et al., 1975), but thought that the oak had been sourced locally. A decade later, when reference chronologies had been developed in Poland, it was realized that the oak had a south-eastern Baltic origin (Eckstein et al., 1986; Eckstein and Wrobel, 2007; Elzanowska et al., 2023). Since then, the dataset of reference chronologies has been growing continuously, and nowadays it covers large areas of Europe. The Baltic datasets for oak are especially well developed and contain thousands of measurements that have been obtained from panel paintings dating from the 15th to the mid-seventeenth century (Daly et al., 2022; Fraiture, 2009; Haneca et al., 2005; Hillam and Tyers, 1995).

While the reference chronologies used by Klein in the 1990s are not mentioned in his reports, the fact that he concluded that the wood had a Baltic origin implies that he must have used chronologies that represented the Baltic region and that were available at their laboratory of Hamburg University. These chronologies could have been the ones developed from Netherlandish and Dutch paintings two decades earlier by Bauch, Eckstein, and himself (Bauch et al., 1972; Elzanowska et al., 2023; Eckstein et al., 1975), or by Fletcher in England (Fletcher, 1977). Since the Hamburg chronologies (NL Gesamt, covering the period 1115–1643 CE; NL Sud, 1173–1619; NL Nord, 1199–1635) were made available in the 1990s to this author’s institution, they were used in this study to reproduce Klein’s results.

#### Software used for the reproduction

While Klein likely used the DOS-based software CATRAS (Aniol, 1983) to carry out the comparison of the tree-ring series from the paintings with the reference chronologies (procedure known as crossdating (Douglass, 1941), the software PAST4 v. 4.3.102 (Knibbe, 2009) was used in this study. In CATRAS, ring-width values from panel paintings that had been annotated in a paper sheet could be inputted manually, to be compared afterwards with the reference chronologies automatically. Then, lines of crossdating results would appear on the computer screen moving upwards, and the dendrochronologist proceeded to write down the matches that seemed promising. Lastly, the tree-ring series obtained would be printed with a plotter, and would be compared on a light table with the chronology providing the best match, which was also plotted on paper. In the late 1990s and early 2000s, several dendrochronology programs were developed with functionalities that allowed the automatic crossdating of numerous tree-ring series and the export of the results in user-friendly text formats, considerably reducing the time required for the crossdating procedure. The use of a different software, however, should not alter the dating results when crossdating tests are used with rigurous criteria (see below).

The tree-ring series developed by Klein from the portraits were compared with the Baltic reference chronologies NL Gesamt, NL Sud, and NL Nord, and with the art-historical chronologies published by Fletcher (1977) on the reported position. Then, the measurement from the GNM panel was compared with the one from the RM painting (the tree-ring series from the RM panel was made available for this study by the RKD). The Student’s *t*-value calculated after normalizing the data with an algorithm developed by Baillie and Pilcher (1973) (hereafter TBP) was used as a reference statistical test, selecting the “Exact” option in PAST4 TTestAlgorithm settings. In the 1980s, TBP values over 3.5 for an overlap of 100 rings or higher were already considered potential matches, with the visual inspection being a decisive step to assign the date (Baillie, 1982). Additionally, the percentage of parallel variation (%PV, first described by Eckstein and Bauch, 1969), was also considered to assess the matches. The %PV indicates the percentage of times that the investigated tree-ring series and the chronology vary up-and-down synchronously. PV values above 63%, with an associated significance level higher than 95.0% (*p* < 0.05), could be considered as a good match. Nowadays, higher TBP values (above 5, for example) are expected, while the good visual match is still a requirement. See Supplementary Information for definitions and additional descriptions of the tests used.

### Conceptual replication implementing state-of-the-art methods

The aim of the second part of the study was to assess whether Klein’s results (including the conclusion that the GNM panel derives from the same tree as that of the RM, attributed to the circle of Rembrandt) could be replicated using state-of-the-art methods. Therefore, both paintings, as well as the match with the Rijksmuseum one, were re-examined, implementing techniques and reference datasets that were not available at the time Klein carried out his dendrochronological study.

#### Inspection and description of the panels

**Fig. 3:**
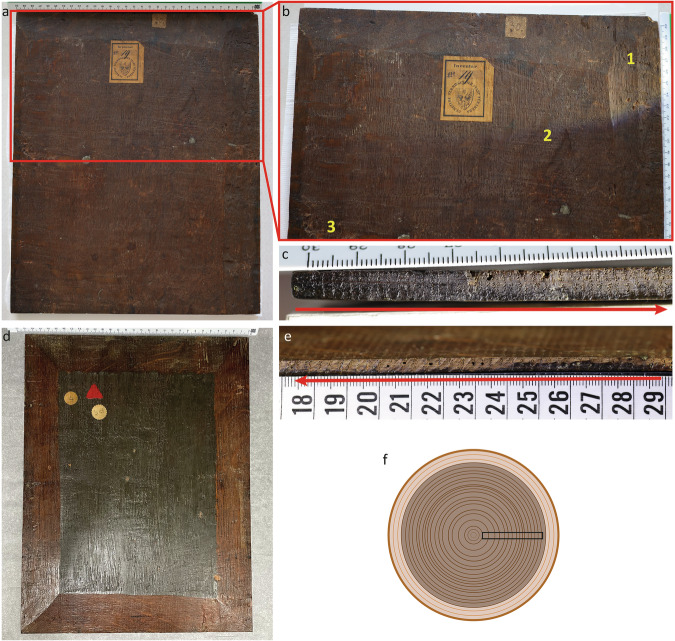
Tool and processing marks on the GNM and MH panels. **a** Reverse of the GNM panel, with bevelled edges on the top, right and partially on the left side, and sharp edge on the bottom; **b** detail of the top-half portion of the board, where different traces can be observed: (1) scraping-plane, (2) handsaw marks, and (3) splitting marks; **c** detail of the transverse section (end grain) at the bottom of the board, showing that it was processed radially from the stem of the tree; **d** reverse of the MH panel where the single oak board and the bevelling can be observed; **e**, **f** this board was also processed radially, as illustrated on the diagram. The arrows indicate the growth direction. Photos: the author.

The GNM panel consists of a single board of oak (*Quercus* sp.) placed vertically (Fig. 3a, b). The board was processed radially from the stem of the tree (Fig. 3c). While the edges are clearly bevelled on the top part and on the right side of the panel (references to sides are done from the perspective of the reverse of the painting), the left part is only slightly bevelled towards the top, and the bottom part has a neat flat surface. This suggests that the panel was trimmed at the bottom and on the left side at some point in time (possibly before being used for this painting). Scraping-plane marks are visible on the right-side bevel. Irregular saw marks (likely resulting from hand-sawing) are also visible on the right half of the board, while signs of splitting can be observed on the left part of the panel. Pith and sapwood are absent.

The MH panel also consists of a single board of oak placed vertically (Fig. 3d). All four edges are bevelled. The board has been processed radially from the stem of the tree (slightly off from the perfect radial cut) (Fig. 3e, f). No evidence of tool traces has been observed.

#### Measuring of tree rings and crossdating

On both paintings, the dendrochronological research was carried out along the transverse section at the bottom of the boards. A brush was used to clean the dust along the surface to be researched. Initially, no preparation with scalpel blades was required to improve the visualization of the tree rings because (i) the transverse section of the GNM panel was smooth enough to visualize the ring boundaries by the naked eye with the aid of lighting, and (ii) the transverse section of the MH panel had already been prepared by Klein (Fig. 3e). However, it was noticed that the outer rings on the GNM version were not clearly visible. Therefore, in consultation with Oliver Mack (head of the Institute for Art Technology and Conservation at the Germanisches Nationalmuseum and chief restorer), and considering that measuring accurately until the outermost ring was essential for this study, it was decided to clean slightly with sharp blades that portion of the surface (approximately 1 cm in length).

Tree rings were photographed with a macro lens, and ring widths were measured on screen with CooRecorder v9.0.1, April 19, 2017 (Larsson, 2017). The photographs included a ruler to allow the calibration of the measurements. Therefore, the obtained ring widths represent absolute values. Crossdating was done in PAST4 v. 4.3.102 (Knibbe, 2009).

## Results

### Results of the reproduction with the 1990s data

#### Reproduction of dendrochronological dates with the original Klein’s data

**Fig. 4:**
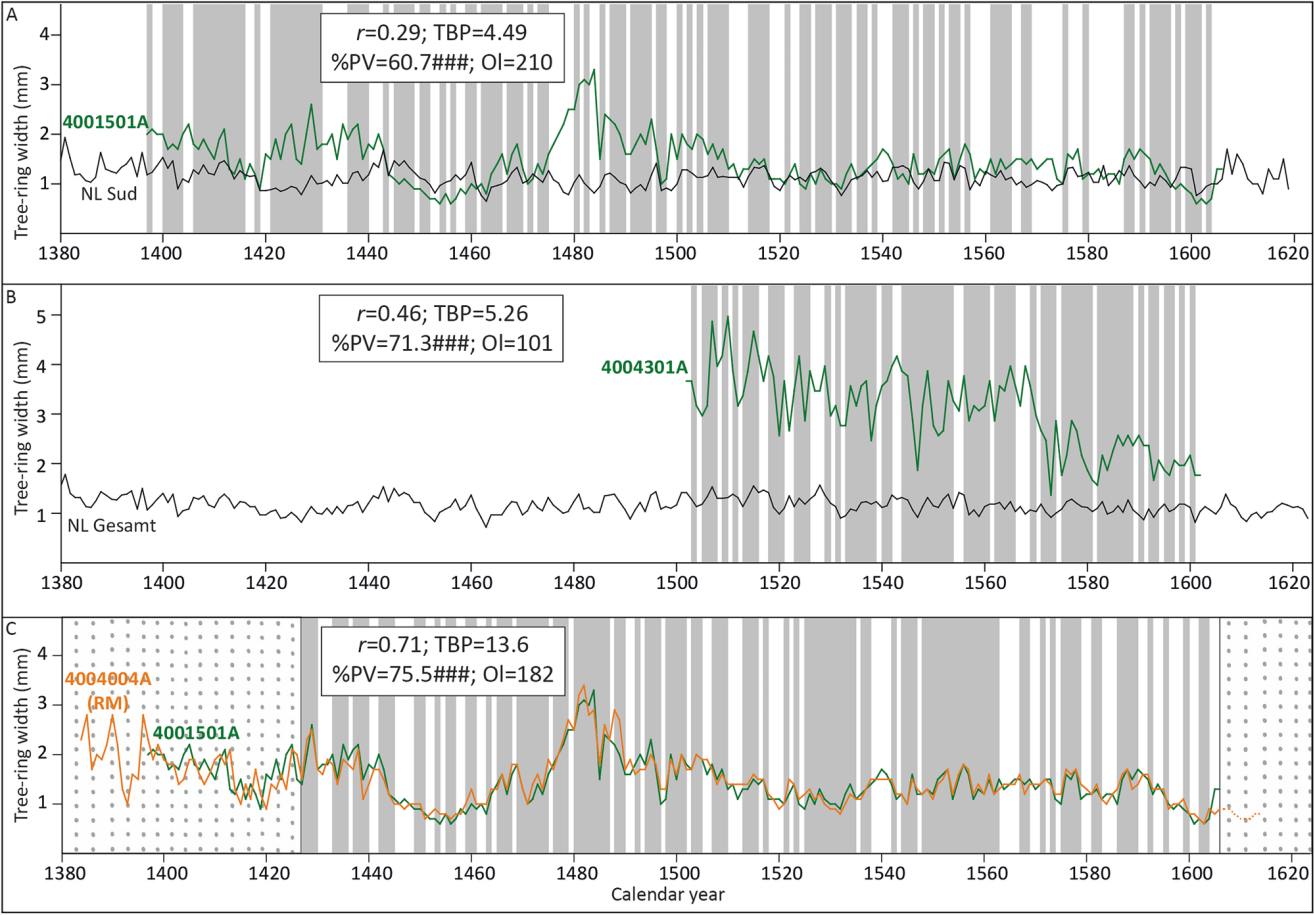
Visual and statistical crossdating results of the reproduction part of this study. Match between the tree-ring series developed by Klein from **A** the GNM panel (4001501A), and **B** the MH panel (4004301A) with the reference chronologies NL Sud and NL Gesamt, respectively. **C** Match between the tree-ring series of the GMN and the RM (4004004A) panel. The dash part of the RM series represents the sapwood rings. The early part of the GNM series (from year 1397 until 1427) has been disregarded to compute the statistical crossdating. Such high statistical values, together with the goodness of the visual match, confirm that both panels originate from the same tree. *r*: correlation coefficient; TBP: Student’s *t* value according to Baillie and Pilcher (1973); %PV: percentage parallel variation as defined by Eckstein and Bauch (1969); ## and ###, significance level of %PV at *p* < 0.01 and *p* < 0.001, respectively; Ol overlap. The shaded area represents the agreement in the parallel variation between the compared series.

The comparison of the tree-ring series developed by Klein with the Baltic reference chronologies developed by the Hamburg laboratory and Fletcher (1977) on the position reported by Klein returned a weak statistical result for the GNM painting (dendro keycode 4001501A) with the NL Sud chronology (TBP = 4.49; Fig. 4A), and with the NL Gesamt (TBP = 4.39), whereas the comparison with the NL Nord and the Fletcher (1977) chronologies delivered TBPs below 4. Considering the length of the series (210 rings), higher statistical values could be expected, but the statistical and visual match with the NL Sud and NL Gesamt chronologies must have been considered acceptable by Klein at the time. This weak result could be the consequence of the inaccuracy of measuring with a hand-lens, although it could also indicate the presence of errors in the beginning or the end of the tree-ring series. It should also be noted that it is possible that Klein used other reference chronologies available at his laboratory, which may have provided better statistical and visual matches. In the case of the MH panel (Klein’s dendro-code 4004301A), the results with the NL Gesamt chronology (TBP = 5.26; Fig. 4B) are acceptable and reasonable for a tree-ring series of that length (101 rings).

#### Reproduction of the match between the tree-ring series of the GNM and the RM panels

The comparison of the tree-ring series of the GNM and the RM panels returned an excellent visual and statistical match for most of the overlapping portion between the measurements (Fig. 4C). Such a good match indicates indeed that the wood of the panels originates from the same tree. However, an error in one of the tree-ring series (likely the one corresponding to the GNM panel, given the relatively low statistical values with the reference chronology) also became evident. The series of the GNM panel seems to have one ring too much (either the ring corresponding to the year 1426 or 1427 was measured or annotated twice) and one too few. This explains the lower-than-expected statistical match of the GNM series with the reference chronologies. Nonetheless, the conclusion that both panels originate from the same tree seems to be correct.

#### Interpretation of felling dates and earliest production dates

Given that the end dates of the tree-ring series created by Klein have been reproduced, the estimated dates for the felling of the tree and the production of the painting should remain the same when the same sapwood statistics are used. However, the following two points must be noted:

- Since the GNM panel originates from the same tree as the panel from the Rijksmuseum painting SK-A-3934, and the latter has eight sapwood rings, their estimated felling date for the tree (i) should be the same (as they are in Klein’s reports), and (ii) should be presented as an interval (Klein presents only the lower—oldest—year of the interval, and the year corresponding to the median, without indicating the upper—youngest—one). Again, the sapwood statistics from Poland used by Klein (namely, 9–24 sapwood rings) are employed here for the estimations. Given that the last ring present in the panel GNM (dated to 1606) is presumed to be the last heartwood ring, we can estimate that the tree was cut between 1615 and 1630 with a 90% probability (Table 2). Adding a minimum of 2 years to account for the seasoning of the wood (as done by Klein), the *most likely production time of the painting* would be between 1617 and 1632.

**Table 2:** Estimated dates for the felling of the trees and the production of the paintings reproducing Klein’s results.

Painting	Klein’s dendro-code	*N*	Begin date	End date	Estimated felling date	Estimated production date
Germanisches Nationalmuseum Nuremberg (GNM)	4001501A	210	1397	1606	Between 1615 and 1630	Between 1617 and 1632
Mauritshuis The Hague (MH)	4004301A	101	1502	1602	After 1611	After 1613

*N*, number of measured rings.
- The MH panel lacks sapwood, which implies that the estimated felling date for the tree must be presented as a *terminus post quem* (date after which the tree was cut, or earliest possible felling date, as expressed in Klein’s reports). Since the minimum number of sapwood rings in the statistics from Poland is nine, adding this number of years to the date of the outermost heartwood ring will allow expressing the earliest possible estimated felling date as a *terminus post quem*. Consequently, the estimated felling date for the tree should be *after* 1611, which would place the earliest possible production time *after* 1613 (Table 2).

### Results of the replication using current methods

#### Comparison of the new measurements with Klein’s measurements

**Fig. 5:**
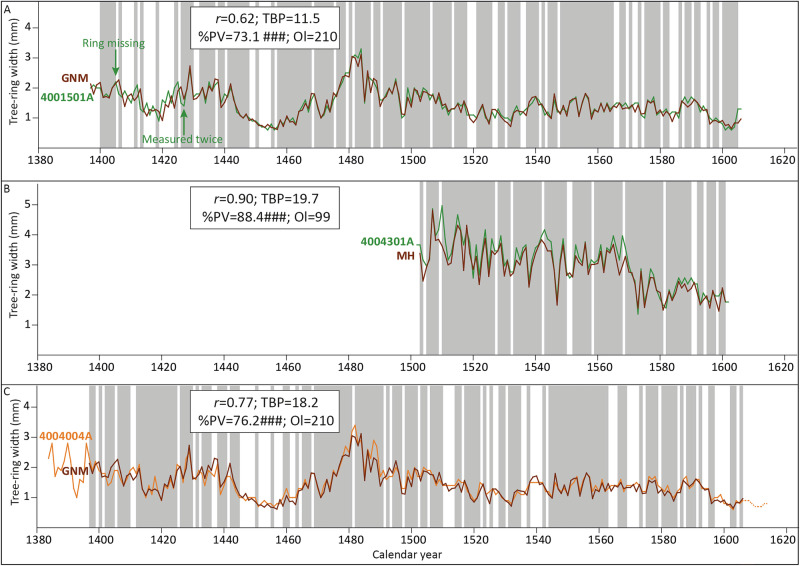
Visual and statistical results of the replication part of the study. **A** Comparison of the tree-ring series from the GNM panel produced by Klein (4001501A, in green) with the one developed by the author for this study (GNM, in brown). **B** Comparison of the tree-ring series from the MH panel produced by Klein (4004301A, in green) with the one developed by the author (MH, in brown). **C** Comparison of the new tree-ring series of the GNM panel (in brown) with the one from the RM panel (4004004A, in orange). These panels originate from the same tree. *r*: correlation coefficient; TBP: Student’s *t*-value according to Baillie and Pilcher (1973); %PV: percentage parallel variation as defined by Eckstein and Bauch (1969); ###, significance level of %PV at *p* < 0.001; Ol overlap. The shaded area represents the agreement in the parallel variation between the compared series.

A measurement series containing 210 tree rings was obtained from the GNM panel, which coincides with the number of rings measured by Klein. This implies that the new tree-ring series of the GNM panel covers the same period as the one measured by Klein (1397–1606). The new measurement was visually compared to those produced by Klein. No significant variation in the amplitude between the series can be observed (Fig. 5A), which is remarkable considering that Klein measured with a hand-lens of 0.1 mm accuracy. However, this exercise has allowed the correction of the measuring errors identified in the original series by Klein of the GNM panel. While both series have the same number of tree rings, it has become evident that the ring corresponding to the year 1405 (or 1406) is missing in Klein’s series, and that the ring corresponding to the year 1427 was measured twice.

The panel MH delivered a series with 99 rings, which is two rings shorter than the sequence measured by Klein, covering the period 1503–1601. The new tree-ring series is shorter by one ring at the beginning and one at the end (Fig. 5B). Those rings have not been measured yet because they are not complete in the transverse section. The ring measured by Klein corresponding to the year 1602 only has earlywood (Fig. S1A in Supplementary Information), and the one corresponding to the year 1502 only has latewood, and the full earlywood is missing (Fig. S1B in Supplementary Information). Therefore, it is not possible to observe their exact width on the transverse section, and they have been left unmeasured.

#### Comparison of the new measurement from the GNM panel with the RM panel

The comparison of the new tree-ring series from the GNM panel with the RM painting has now returned an outstanding visual and statistical match (Fig. 5C). Such an excellent fit confirms that both panels very likely originate from the same tree. If this is the case, the outermost measured ring present in the GNM panel likely corresponds to the heartwood/sapwood border on the RM painting, which justifies the estimation of the felling date of the tree within a closed range of years.

#### Crossdating with reference chronologies

**Fig. 6:**
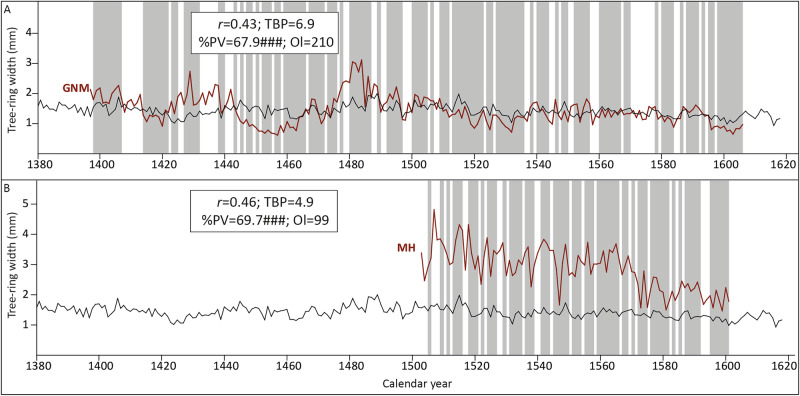
Results of the replication study. Visual and statistical match between **A** the new tree-ring series of the GNM panel and **B** the GH panel with the reference chronology 2021BLT2 (black). *r*: correlation coefficient; TBP: Student’s *t* value according to Baillie and Pilcher (1973); %PV: percentage parallel variation as defined by Eckstein and Bauch (1969); ###, significance level of %PV at *p* < 0.001; Ol overlap. The shaded area represents the agreement in the parallel variation between the compared series.

Since the new measurements from both panels are already dated through crossdating with the measurements by Klein, the goal of the comparison with currently available reference chronologies was to find out the provenance of the wood, in order to determine the sapwood statistics to be used to estimate the felling date of the trees. Crossdating with reference chronologies returned the best match for the GNM panel with a Baltic chronology by Daly and Tyers (2022) representing Poland (2021BLT2; Fig. 6A). The MH panel, however, does not show a distinctly strong match with any particular reference chronology to allow determining the provenance of the wood. While the best match is obtained with a Baltic chronology by Daly and Tyers (2022) representing the southeast of Lithuania (2021BLT3), the statistical match is rather weak (TBP = 5), and closely followed by the results with the 2021BLT2 chronology representing Poland (TBP = 4.9). Furthermore, other chronologies from the east of the Netherlands/northwest of Germany replicate the date obtained for this MH panel, which suggests that the wood likely originates from the southern rather than the eastern Baltic. Therefore, the sapwood statistics for Poland have been used to infer the felling date of the tree (Table 3). The Polish chronology 2021BLT2 by Daly and Tyers (2022) has been used in the visual comparison for assessment purposes (Fig. 6B).

**Table 3:** Estimated dates for the felling of the trees produced in this study.

Painting	DR dendro-code	*N*	Begin date	End date	Estimated felling date^a^	Earliest possible production date	Likely production date
Germanisches Nationalmuseum Nuremberg (GNM)	40560011	210	1397	1606	Between 1615 and 1630	Between 1617 and 1620	Between 1617 and 1635
Mauritshuis The Hague (MH)	40550011	99	1503	1601	After 1610	Between 1612 and 1615	After 1612

^a^Based on sapwood statistics for Poland reported by Wazny (1990) for the 90% confidence interval.

#### Interpretation of felling dates and earliest possible production dates

Given that the RM panel has sapwood and that the GNM panel originates from the same tree, it has been possible to determine that the outermost ring in the GNM panel corresponds to the heartwood/sapwood border. Therefore, the felling date of the tree can be estimated within a range of years. Given that the wood originates from Poland, sapwood statistics for this geographical area produced by Wazny (1990) should be used to estimate the felling date of the tree. Considering that trees in this area have between 9 and 24 sapwood rings, the felling date of the tree can be estimated to have occurred between 1615 and 1630 (Table 3). To infer when the painting could have been made, some years must be accounted for in the transport and seasoning of the wood. From observations of panel paintings signed by the artists and retaining partial sapwood, Klein et al. (1987) proposed that the seasoning time was about 2 years in the seventeenth century. Subsequently, Wadum (1998) advised considering up to 5 years. Those numbers would place the *earliest possible* production period between 1617 and 1620, with the *likely* production time of the painting being between 1617 and 1635 CE (Table 3).

The MH panel lacks sapwood, and it is not possible to know how many rings are missing until the heartwood/sapwood border. Therefore, only a *terminus post quem* can be provided for the felling of the tree. Considering a provenance in the southern Baltic, the sapwood statistics proposed by Wazny (1990) for Poland should be used, placing the estimated felling date of the tree *after* 1610. In this case, the *earliest possible* production date for the painting would be between 1612 and 1615, with a date *after* 1612 being *most likely* (Table 3).

## Discussion

Dendrochronology is often used to validate or to test the attribution of panel paintings (Bernabei et al., 2023; Brookhouse et al., 2020), sculptures (Domínguez-Delmás et al., 2021b), and string instruments (Bernabei, 2021; Cherubini et al., 2022; Soltani et al., 2021). However, replication studies like the one presented here are very scarce (see one other example by Elzanowska et al., 2023). In this study, the reproduction and replication of the dendrochronological research of the panels of two portraits of a young Rembrandt carried out in the 1990s have confirmed the dates for the wood reported by Klein in the 1990s. In the reproduction part of this study, two measuring errors were detected in the original measurement by Klein of the GNM panel. In the early part of the tree-ring series, a ring was missing, and another one had been annotated twice. The net result was just a mismatch of part of the tree-ring series, and since the misalignment occurred in a small portion of a long tree-ring series and in the early years, the consequence was a weak statistical match obtained for the GNM version. Should these measurement errors have occurred towards the center of the series or have covered a longer span, the tree-ring series would have likely remained undated or potentially misdated, unless the right position had been identified by visual crossdating. These measurements errors could have happened in different steps of the process: during the recording of the tree rings with a hand-held lens, when a person would read aloud the ring values and a second person would write them down on a paper sheet (the observer could fail to identify all rings correctly, or the person annotating the values could miss one, or annotate one double), or during the insertion of the ring-width values in the DOS-based software CATRAS, available at the time, as they had to be typed in manually. These different steps, potentially involving several people, made the method susceptible to delivering tree-ring series with errors. Consequently, the re-examination of paintings originally researched by hand-held lens, now using state-of-the-art methods, is serving to identify and correct previous measuring errors that in some cases led to spurious dating results (e.g., Domínguez-Delmás et al., 2021a).

The replication using modern methods allowed identifying the exact positions of the faulty rings. The corrected tree-ring series shows an outstanding match with the one from the RM panel, further supporting that both panels originate from the same tree, as initially proposed by Klein. The replication has also revealed that while the measuring method employed by Klein in his 1990s studies had an accuracy of 0.1 mm at best (Bauch et al., 1972), the measurements produced have a negligible difference in magnitude to those produced through measurements on digital photographs. This is an interesting insight, as it implies that measurements from art historical material obtained with a hand-held magnifying glass can be used for studies that rely on absolute ring widths, such as forest development in past centuries (e.g., Muigg et al., 2020; Muigg and Tegel, 2021).

Fortunately, the hand-held lens method has been widely abandoned now, and most dendrochronologists have been using digital photography to register the tree-ring patterns on artworks since the early 2000s (Edvardsson et al., 2021; Fraiture, 2014, 2002; Fraiture and Haneca, 2018; Seim et al., 2024; Wadum et al., 2022). Some have even explored the implementation of non-invasive methods through X-ray-based techniques, such as computed tomography (Daly and Streeton, 2017; Domínguez-Delmás et al., 2021a, 2021b), or line-trajectory tomographic techniques (Bossema et al., 2021). The use of digital photographs or X-ray-based imaging allows not only the re-examination of the measurements multiple times without the need to go back to the painting, but also the replication of the research by different experts. This requires the images to be made openly available, a practice that must be supported (and perhaps also required) by the museums and/or private collectors owning the artworks, and by the dendrochronologists carrying out the research.

The availability of reference chronologies is also crucial for the accurate reproduction of dendrochronological studies. Klein’s reports consisted generally of a letter in which only basic information was provided, such as the number of boards making up the panels, the number of tree rings in the researched boards, the date found for the tree-ring series, the provenance of the wood, the estimated felling date of the tree, and the likely production time of the paintings. Unfortunately, no information about the chronology, providing the date, or the statistical values supporting the date was registered, hindering the strict reproduction of the study. When dating reports only provide a date without additional supporting information, one can only guess the chronologies that may have been used (based on information regarding the provenance of the wood), and accepting the result becomes an act of faith that reduces the science to an opaque black box. This practice is nowadays abandoned by most dendrochronologists, and dating reports typically include tables and graphs with statistical values and other information that allow assessing how the date was obtained (see, for example, openly accessible dendrochronological reports in DataverseNL https://dataverse.nl/dataverse/dccd, or in Zenodo.org). Such reporting standards were already proposed by Miles (1997) and Hillam (2004), and together with the open accessibility of the data in those general repositories, or in dedicated ones such as the Dendro4Art database managed by the RKD, are the best way forward for dendrochronology to comply with FAIR principles (findable, accessible, interoperable, and reusable).

While not being openly accessible, the reference chronologies developed by the Hamburg tree-ring laboratory in the 1970s and 1980s were lent to the Cultural Heritage Agency of the Netherlands in the 1990s, when dendrochronology was being developed in the Netherlands (Jansma, 1995). The exchange of reference chronologies and tree-ring data is still a common practice between European dendroarchaeologists, and while most private laboratories are still reluctant to make their data and/or metadata openly available (Jansma et al., 2012), they are keen to share it on demand for scientific purposes (e.g., Ljungqvist et al., 2022).

Lastly, the estimation of felling dates for the trees and potential production dates for the paintings has been revised in the replication part of this study. Based on the exact dendrochronological dates for the outermost rings, the sapwood statistics have been applied rigorously, reporting the one for the GNM panel as an interval, given that the outermost ring corresponds to the heartwood/sapwood border, and the one from the MH panel as a *terminus post quem*, given that the outermost ring is a heartwood one. The estimation of potential production dates for the paintings has now been made considering the 2 years reported by Klein et al. (1987), but also the up to 5-year observation reported by Wadum (1998). The latter observation was not available at the time when Klein carried out the dendrochronological study of these portraits, which possibly explains why he did not account for it in his reports. This highlights the relevance of the continuous expansion of tree-ring datasets (from both historic timbers and living trees), as the development of region-specific chronologies and sapwood observations allows pinpointing the provenance of the wood more accurately. By inference, more refined felling dates for the trees can be estimated, which results in more precise estimations of the production time of artworks.

## Conclusions

This study demonstrates that dendrochronology is a well-established science that should always return the same outcome if used rigorously. As long as the tree-ring sequences are recorded correctly and reference chronologies for the source area are used, the research should deliver the same dating outcome regardless of the recording method or software employed. However, it also highlights the need for detailed reporting that includes mention of the datasets used and the statistical values that support the match with reference chronologies. Only then, and provided that the tree-ring data produced and the reference chronologies are openly accessible or accessible on demand, can reproduction studies be carried out rigorously.

Similarly, the long-term storage and preservation of tree-ring data and source material (e.g., photographs of the transverse sections of boards where the tree rings have been measured) is paramount to allow rechecks to identify potential measuring errors, and/or re-examinations by different dendrochronologists, without having to re-examine the painting again. In this regard, the development of the Dendro4Art database by the RKD and the National Gallery of Denmark to host Klein’s legacy data and reports was a major step forward towards FAIR principles in art-historical dendrochronology, and to enable the long-term preservation of images, dendrochronological data, and metadata derived from current and future research of artworks. Other platforms, such as the earlier-mentioned DataverseNL and Zenodo, can also be used as repositories for the long-term storage of the reports, tree-ring data, and photographs derived from dendrochronological research. Therefore, it is advised that museums and art collectors commissioning dendrochronological research request dendrochronological reports that comply with international standards, as well as the shared stewardship of the tree-ring datasets and digital images of their artworks.

## Supplementary information

Supplementary information

## Data Availability

Klein’s tree-ring series from the 1990s can be requested from the Netherlands Institute of Art History, referring to the persistent identifiers of the artworks: https://rkd.nl/technical/5008997 (that of GNM), https://rkd.nl/technical/5005287 (that of MH), and https://rkd.nl/technical/5009945 (that of the RM painting SK-A-3934). The two tree-ring series created in the replication part of this study, after re-examination of the paintings, can be found in DataverseNL (10.34894/HLYEJS). All openly accessible additional material related to the ‘Replicating a Rembrandt Study’ is stored on OSF|Replicating a Rembrandt Study.

## Footnotes

1 RKD persistent identifiers for the reports: for the GNM painting: https://rkd.nl/technical/5008997; for the MH painting: https://rkd.nl/technical/5005287; for the RM painting https://rkd.nl/technical/5002600.
