# Supplementary material for: A replication study in dendrochronology—revisiting the panels of two portraits of Rembrandt
Source: Humanit Soc Sci Commun. 2025 Nov 19;12(1):1778. doi: 10.1057/s41599-025-06066-2 (PMC12629980; doi:10.1057/s41599-025-06066-2)
Supplement: Supplementary file 1 — Supplementary information [file 41599_2025_6066_MOESM1_ESM.pdf]

Supplementary information

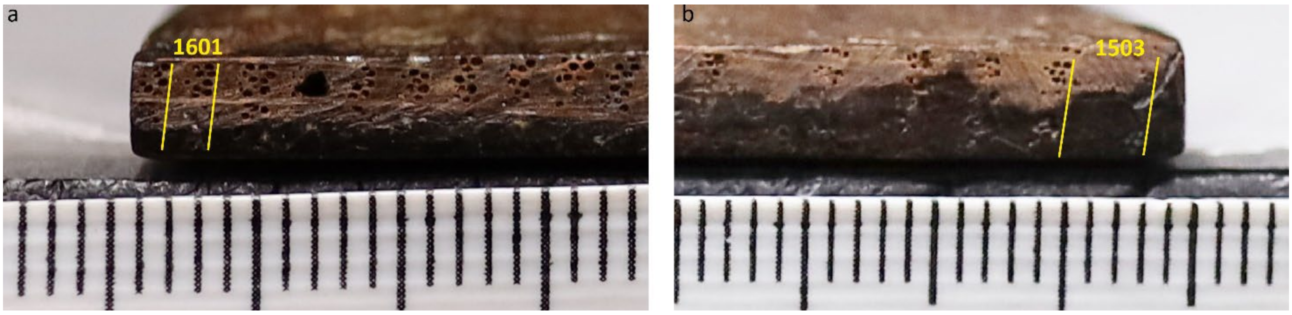

Figure S1. Years corresponding to the most recent (a) and oldest (b) complete rings measured on the MH panel. The most external rings in the panel are not complete and have been left unmeasured. Photos: the author.

Glossary and abbreviations

N	Total number of measured rings in the sample;
Begin date	Date of the first ring (closest to the pith of the tree) measured in the sample;
End date	Date of the last ring (most recent ring, closest to the bark of the tree) measured in the sample;
Estimated felling date	Date of the last ring plus the estimated mean number of rings to the bark edge when the WK is not present;
r	Pearson correlation coefficient;
TBP	Value of the Student *t*-test according to (Baillie and Pilcher, 1973); this value is used to identify the match between two tree-ring series for which the correlation reaches its highest value. Student's *t* values over 6 for an overlap of 100 rings are likely to indicate a match. To determine the provenance of the wood, TBP values above 7 should be considered, together with the areas represented by chronologies providing replications of the date.
%PV	Percentage of parallel variation (Eckstein and Bauch, 1969); this value indicates, for the overlapping period between two tree-ring series, the percentage of years in which the ring-widths increase or decrease similarly. Values higher than 63%, for an overlap of 100 rings are highly significant and indicate a match;
Overlap (Ol)	Number of overlapping rings between two curves in their matching position;
Reference chronology	Chronology used to date the sample.

References

Baillie, M.G.L., Pilcher, J.R., 1973. A simple crossdating program for tree-ring research. Tree-Ring Bull. 33, 7–14.

Eckstein, D., Bauch, J., 1969. Beitrag zur Rationalisierung eines dendrochronologischen Verfahrens und zu Analyse seiner Aussagesicherheit. Forstwissenschaftliches Cent. 88, 230–250.
